# Enhancing touch sensibility by sensory retraining in a sensory discrimination task *via* haptic rendering

**DOI:** 10.3389/fresc.2022.929431

**Published:** 2022-08-01

**Authors:** Eduardo Villar Ortega, Efe Anil Aksöz, Karin A. Buetler, Laura Marchal-Crespo

**Affiliations:** ^1^Motor Learning and Neurorehabilitation Laboratory, ARTORG Center for Biomedical Engineering Research, University of Bern, Bern, Switzerland; ^2^Division of Mechanical Engineering, Department of Engineering and Information Technology, Institute for Rehabilitation and Performance Technology, Bern University of Applied Sciences, Burgdorf, Switzerland; ^3^Department of Cognitive Robotics, Delft University of Technology, Delft, Netherlands

**Keywords:** haptic rendering, sensory rehabilitation, active exploration, passive exploration, touch, texture discrimination

## Abstract

Stroke survivors are commonly affected by somatosensory impairment, hampering their ability to interpret somatosensory information. Somatosensory information has been shown to critically support movement execution in healthy individuals and stroke survivors. Despite the detrimental effect of somatosensory impairments on performing activities of daily living, somatosensory training—in stark contrast to motor training—does not represent standard care in neurorehabilitation. Reasons for the neglected somatosensory treatment are the lack of high-quality research demonstrating the benefits of somatosensory interventions on stroke recovery, the unavailability of reliable quantitative assessments of sensorimotor deficits, and the labor-intensive nature of somatosensory training that relies on therapists guiding the hands of patients with motor impairments. To address this clinical need, we developed a virtual reality-based robotic texture discrimination task to assess and train touch sensibility. Our system incorporates the possibility to robotically guide the participants' hands during texture exploration (i.e., passive touch) and no-guided free texture exploration (i.e., active touch). We ran a 3-day experiment with thirty-six healthy participants who were asked to discriminate the odd texture among three visually identical textures –haptically rendered with the robotic device– following the method of constant stimuli. All participants trained with the passive and active conditions in randomized order on different days. We investigated the reliability of our system using the Intraclass Correlation Coefficient (ICC). We also evaluated the enhancement of participants' touch sensibility *via* somatosensory retraining and compared whether this enhancement differed between training with active vs. passive conditions. Our results showed that participants significantly improved their task performance after training. Moreover, we found that training effects were not significantly different between active and passive conditions, yet, passive exploration seemed to increase participants' perceived competence. The reliability of our system ranged from poor (in active condition) to moderate and good (in passive condition), probably due to the dependence of the ICC on the between-subject variability, which in a healthy population is usually small. Together, our virtual reality-based robotic haptic system may be a key asset for evaluating and retraining sensory loss with minimal supervision, especially for brain-injured patients who require guidance to move their hands.

## 1. Introduction

Stroke is the most common acquired brain injury that causes persisting and long-term disability in adults (1). Between 65 and 85% of stroke survivors suffer from somatosensory impairment (2, 3), hampering individuals' ability to interpret somatosensory information (4), and thus, their ability to perform skillful movements independently (5, 6). Importantly, somatosensory impairment increases patients' hospitalization time (7) and limits the recovery of sensorimotor function (8). Despite the negative impact of somatosensory impairment on upper limb functionality and recovery (9, 10), somatosensory training is not the standard of care following stroke (5, 11) and generally receives less attention than motor training (9). The lack of time for therapy and limited access to somatosensory training guidelines are some factors that may contribute to the lack of attention to sensory rehabilitation (4).

Somatosensory interventions are therapeutic techniques performed by a therapist designed to retrain sensory function (12). Somatosensory interventions can be classified as sensory retraining—i.e., interpretation of a stimulus—and sensory stimulation –i.e., afferent stimulation (13). **Sensory retraining** involves the patients' interpretation of stimuli, which are usually provided by the therapist. An example of sensory retraining intervention is the tactile discrimination test (TDT), a conventional approach to evaluate and train touch sensibility in clinical settings (2). TDT is performed by asking the patient to tactually explore gratings textures, also known as active touch. The therapist may also guide the patient's paretic hand (i.e., passive touch) when the patient has a severe motor deficit. **Sensory stimulation**, by contrast, relies on the therapist providing a stimulus, e.g., transcutaneous electrical nerve stimulation, while the patient does not move and is asked to simply feel the stimulus without an active motor or cognitive reaction (5, 9).

Somatosensory interventions have shown promising results in enhancing sensory discrimination—i.e., the skill to discern and interpret specific sensory stimuli (12)—in stroke survivors (6). Moreover, sensory retraining interventions have been found beneficial for the recovery of motor function in stroke patients (9) and improvement of somatosensory function, especially those interventions based on the discrimination of textures, proprioceptive discrimination tests, and tactile object recognition (14–16). Yet, in the last decade, six reviews concluded that there is insufficient empirical evidence regarding the effectiveness of sensory retraining interventions on the recovery of sensorimotor function after a brain injury (5, 6, 9, 11, 13, 17). These reviews cited poor quality of study designs, variations in outcome measurements (18), small sample sizes, and inadequate statistical power to detect meaningful differences between control and treatment (6) as limiting factors to draw clear conclusions. Two recent reviews about the effectiveness of somatosensory interventions concluded that high-quality research is necessary to determine whether sensory retraining is effective in stroke rehabilitation (9, 11).

Quantitative reliable assessment of the sensorimotor performance is needed to evaluate if patients are achieving functional rehabilitation gains after somatosensory interventions (6, 17). However, conventional somatosensory assessments may present variations in results, especially when the assessment is performed by different clinicians (19). A systematic, qualitative, and objective assessment of touch sensibility would facilitate diagnosis, prognosis, and the selection of adequate somatosensory treatments according to the patients' touch sensibility (4).

Robotics is a promising technology to quantitatively assess somatosensory function and provide somatosensory training. Compared to other conventional treatments, robotic devices are capable of delivering precise and reproducible stimuli (20). Further, robots can physically guide the patients' limbs during sensorimotor training (21), facilitating the admission of patients with severe motor impairments into the training and enhancing their motivation and engagement during repetitive and intensive practice (22). However, despite their potential, the usage of robots to assess and treat somatosensory function is, to date, mainly neglected (23, 24). Although research efforts have been made to assess and enhance proprioceptive function with robots—e.g., Kenzie et al. (25), Zbytniewska et al. (20), Elangovan et al. (15), Yeh et al. (16), and Cappello et al. (26)—, fewer efforts have been done into assessing and training tactile function (23). Currently, there is a clinical need to develop robotic systems to assess and train touch sensibility in patients with limited motor function that within this project we aim to meet.

When designing a robotic device for assessment and training of touch sensibility, especially for those patients with severe motor impairments who require robotic assistance to move their paretic limbs, it is important to first understand the differences in touch sensibility perception when a patient is assisted or not. The perception of touch sensibility may differ depending on the mode of touch: active or passive touch. Fundamental studies of touch sensation refer to **active touch** as the *action* of touching, e.g., by actively moving the limbs. In contrast, **passive touch** refers to two different processes: 1) the act of being touched, while the limb does not move/is not passively moved (27), and 2) the act of touching while being assisted by an external agent (e.g., by a therapist) (28). It is not yet fully understood how the nervous system processes active and passive touch. According to Pertovaara et al. (29), active touch relies on the afferent-induced mechanism and the motor command signals, whereas passive touch relies mainly on the afferent-induced mechanism. Further, active touch involves participants choosing their exploration strategy, notably the intention, planning, preparation, and execution of the movements (30). On the contrary, passive exploration is considered to minimize any involvement of decision-making processes (30), allowing participants to focus on the perception of the stimulus (31). Consistently, Van Doorn et al. (30) found an increase in attentional networks activity in the parietal lobe in active touch compared to passive touch using functional Magnetic Resonance Imaging (fMRI).

In their review, Symmons et al. (28) attributed differences in sensory perception between active and passive modalities to the task characteristics and the nature of the stimulus, rather than the exploration mode. For example, Magee and Kennedy (31) found passive exploration to be better in the discrimination of dot-pattern shapes when compared to active exploration, while Richardson et al. (32) found no differences between active and passive touch in discriminating embossed-dots mazes. Vega-Bermudez et al. (33) associated the differences between active and passive touch to two main causes: 1) the experimenter failing to provide equivalent somatosensory information in both modalities and 2) differences in the sensory neural mechanisms underlying tactual pattern recognition behavior. Thus, for comparing active vs. passive touch, the experimenter should provide the same stimulus in both conditions, including kinesthetic information regarding the movement of the limb. Furthermore, other subjective factors such as motivation, might play a role in the differences between passive and active touch. Active engagement during training has been associated with higher motivation (34), while high motivation is associated with an increase in motor adjustments based on sensory signals (35). However, passive touch may allow participants to better focus on the task (i.e., to the sensory input), enhancing their perceived competence (36).

**Figure 1:**
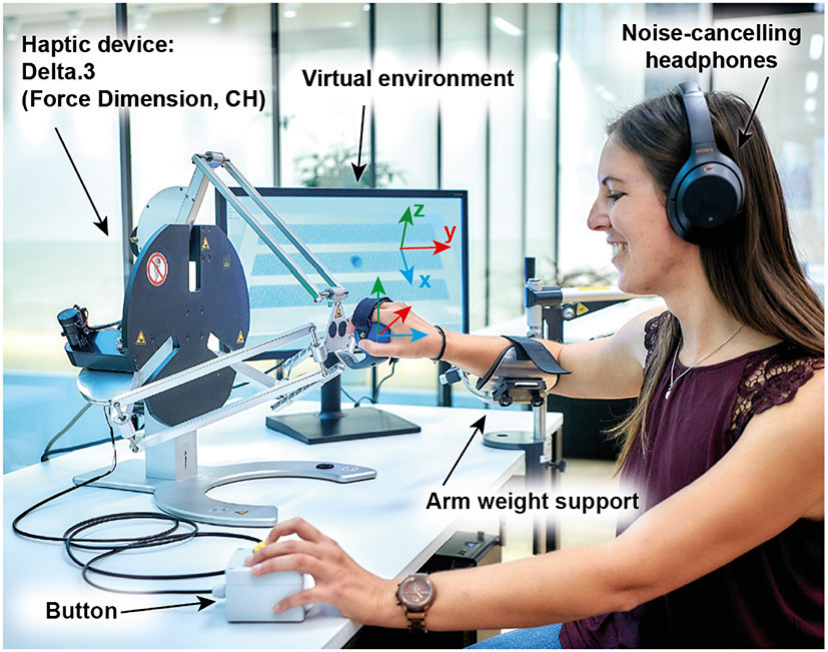
Experimental set-up. The Delta.3 robot was located on a table next to a LED monitor, which showed the virtual environment. Participants wore noise-canceling headphones and used an arm-weight support system, which was adjusted to each participant's individual arm weight. Note that the monitor is located on the right-side of a right-handed user only for illustration purposes (during the experiment it was located on the left side of the robot).

This study aims to evaluate a novel robotic intervention to assess and train tactile function and, when needed, provide robotic assistance to guide the hand during passive touch. We developed a sensory discrimination task to characterize and treat the acuity of touch sensibility *via* the perception of virtual textures rendered by a haptic robotic device (Figure 1). The novelty of our approach relies on the provision of the haptic rendering forces from the virtual textures that are independent of the normal forces that participants exert against the virtual surface, and thus, providing more controlled stimuli within and between participants.

We ran a 3-day within-subject experiment with 37 healthy participants who actively and passively (i.e., assisted by the robot) explored a set of virtual haptically rendered textures and selected the odd texture among three visually identical textures. The first session consisted of two initial baselines. The remaining two sessions comprised three phases: baseline, training, and retention, performed each with passive or active touch in randomized order. In this paper, we evaluated: 1) the system reliability, 2) the change in participants' touch sensibility after somatosensory training, 3) differences in touch discrimination changes pre-post training between active and passive conditions, and 4) differences between passive and active touch conditions on participants' motivation. We hypothesized that passive exploration would have a higher reliability coefficient than active exploration since the provision of stimuli is more controlled. We also hypothesized that participants would improve their tactile acuity of textures after training. Moreover, the improvement of touch sensibility after training would not differ between active and passive conditions in our controlled set-up. Finally, we expected active exploration to generally enhance participants' effort, pressure, and enjoyment/interest during the task (35), yet, haptic guidance may specifically increase the self-reported level of perceived competence (36).

## 2. Methods

### 2.1. Participants

Thirty-seven healthy participants gave written informed consent to participate in the study. One participant was excluded from the analysis due to a hardware failure during the second session and could not participate in the third session. Thus, 36 participants (50% females) completed the experiment, see demographic information in Table 1. The study was approved by the local ethical committee (Swiss Cantonal Ethics Committee; Basec ref: 2018-01179) and the Swiss Agency for Therapeutic Products (Swissmedic ref: 100000432), and conducted in compliance with the Declaration of Helsinki. The participants' hand dominance was assessed with the Edinburgh Handedness Inventory (37).

**Table 1:** Participants demographics.

**Characteristics**	**Participants (*N* = 36)**
	**no. (%)**
**Gender**	
Female	18 (50)
Male	18 (50)
**Age**	
<25 yr	4 (11.1)
25-35 yr	25 (69.4)
≥35 yr	7 (19.4)
**Handedness**	
Right	34 (94.4)
Left	2 (5.6)

*Thirty-seven (one participant excluded in data analysis) participants were recruited for the experiment*.

The sample size was calculated using the R package “sensR” (38). We ran a first pilot experiment with seven healthy participants, in which the average number of correct responses after 40 trials was 25. We then used the average number of correct responses, assumed the desired power of 0.95, a type I error of alpha equal to 0.05, and a probability guess of 1/3 (i.e., triangle test) to compute the sample size. The result of the sample size computation was 34.

### 2.2. Experimental set-up

The experimental set-up (Figure 1) consisted of a 24 inch monitor (S24E650, Samsung, South Korea), a robotic device (Delta.3, Force Dimension, Switzerland), a passive arm weight support (SaeboMAS mini, Saebo, USA), noise-canceling headphones (WH-1000XM4, Sony, Japan), and a custom-made response box with a push-button.

During the experiment, participants were seated at a desk on a comfortable chair with backrest. Their dominant arms were placed using Velcro^®^ straps in a passive arm weight support device attached to the table. The passive arm weight support was used to reduce fatigue during the experiment. The weight compensation level was adjusted to each participants' arm weight and kept constant during the three sessions. The location of the monitor, robot, arm-weight support, and response box were adjusted to the handedness of each participant before the start of the experiment and kept constant during the whole experiment. Participants performed the experiment with their dominant hand. Right- (left-)handed participants had the monitor and response box on the left (right) side of their sagittal plane and the arm-weight support on their right (left) side.

Participants were asked to hold the end effector of the haptic device with their dominant hand at all times. Right- (left-)handed participants had the robot located on the right- (left-)side of their sagittal plane and aligned to the shoulder of the dominant arm. The chair height was adjusted such that the participant's shoulder was not below the robot end effector in the center of the workspace. We exchanged the robot commercial end effector with a new 3D-printed handle to improve participants' comfort. The participants' hands were secured to the robot end effector using a Velcro^®^ strap. The participants were wearing active noise-canceling headphones and we delivered white-noise during the experiment to mask any auditory cues from the robot actuators.

### 2.3. The haptic exploration task

We designed a virtual environment to assess and train participants' touch discrimination using haptically rendered virtual textures (i.e., stimuli) using Unity (Unity Technologies, USA). During the experiment, the participants were asked to discriminate the odd texture among three visually identical textures. We asked participants to select the odd texture by pressing a custom-made button on the response box with their non-dominant hand when they were on top of the texture they believed was different from the other two. If a participant pressed the button outside a texture, we registered the last texture explored as their response.

The virtual textures were displayed horizontally on the monitor. Each texture had the same dimensions in the physical (robot) workspace (0.176 x 0.02 m), an area large enough to allow participants to move the robot end effector tangentially across the texture. The textures were located in parallel and separated 0.01 m from each other along the *x*-axis (blue axis in Figure 1).

**Figure 2:**
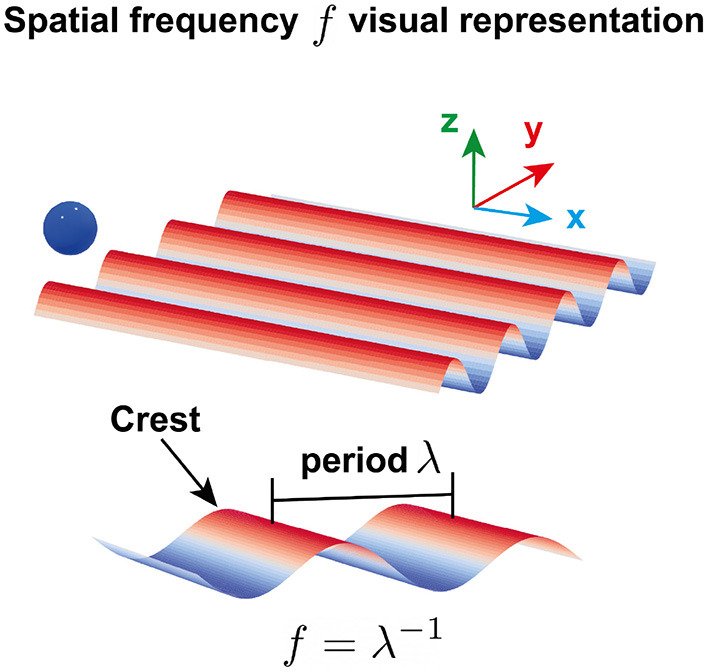
Visual representation of a virtual sinusoidal grating. Each texture consisted of adjacent crests along the *y*-axis. The spatial frequency *f* was defined as the inverse of the spatial period λ, i.e., the distance between two consecutive crests. The blue ball illustrates the position of the robot end effector, *y*_*EE*_, in the virtual environment. The scale and size of the texture and the ball are only for visual purposes and do not represent the scale and size used in the experiment.

We rendered the virtual textures as sinusoidal gratings (see Section 2.5 and Figure 2) using the haptic device Delta.3. During each trial, participants explored the textures either with active or passive touch conditions (see Section 2.6). Participants were allowed to explore each texture as many times as wanted and switch between textures when desired.

### 2.4. Tactile stimuli

The stimuli consisted of virtual sinusoidal gratings along the robot end effector *y*-axis (Figure 2). The interaction forces between the participants' hands and the gratings were rendered by the Delta.3 robotic device and generated by the following equation:


(1) $$F_{g}=C\sin\;(2\pi fy_{EE}).$$

The grating forces *F*_*g*_ depended on the robot end effector position along the *y*-axis, *y*_*EE*_, and the spatial frequency *f* of the grating, i.e., the reciprocal of the spatial period defined as the distance between two consecutive crests (Figure 2). The constant *C* = 3 N determined the amplitude of the sinusoidal and did not change between textures.

Several different virtual textures were generated (see Table 2). They included eight *comparison stimuli* (*Co*) and one *standard stimulus* (*St*), which differed between them in terms of the value of the spatial frequency *f*. The standard stimulus was fixed during the experiment and employed as a basis for quantitative comparison against the set of comparison stimuli, i.e., stimuli with varying physical attributes. In every trial –defined as a single discrimination attempt of the odd texture in a set of three textures– the three virtual textures consisted of two types of stimuli, the *St* and a random stimulus selected from the set of *Co*. We employed the triangle testing method for sensory discrimination [described in Bi (39), page 3], i.e., two of the textures were equal with possible combinations sets: St/St/Co, St/Co/St, Co/St/St, Co/Co/St, Co/St/Co, St/Co/Co. The presentation order of the stimuli followed the method of constant stimuli [described in Gescheider (40), page 46].

**Table 2:** The set of experimental stimuli.

	***f***_*****St*****_ **(*****m***^**−1**^**)**				***f***_*****Co*****_ **(*****m***^**−1**^**)**			
	**More coarse**				**Less coarse**			
164	100	116	132	148	180	196	212	228

*The standard stimulus S_t_ with spatial frequency f_St_ was kept constant during the experiment. The spatial frequency of the comparison stimulus f_Co_ was varied every trial along with the more coarse and less coarse textures sets. Each Co stimulus was presented five times during a block of 40 trials*.

The *St* was fixed through all trials, while the *Co* was varied in each trial from the pool of preselected *Co*. Two preselected pools of *Co* were created, which spanned two ranges of textures: more coarse and less coarse textures (Table 2). The more coarse textures had a spatial frequency that ranged from 100 to 148 *m*^−1^, and the less coarse textures ranged from 180 to 228 *m*^−1^. Each spanned set of the *Co* (i.e., more and less coarse sets) consisted of four different stimuli with equal inter-space distance between consecutive *Co* that was set to 16 *m*^−1^. The most coarse texture (100 *m*^−1^) corresponded to a spatial period of 10 mm, whereas the least coarse texture (228 *m*^−1^) corresponded to a spatial period of 4.38 mm. The *St* was set to 164 *m*^−1^ –the mean of all *Co* spatial frequencies– and kept constant throughout the experiment. We chose the spatial frequency of the *St* to be the average of all *Co* spatial frequencies to avoid any bias toward either of the textures roughness directions.

The *Co* spatial frequency values in Table 2 had various levels of discrimination difficulty, i.e., the closer they were to the *f*_*St*_ the more similar they were perceived and more difficult to differentiate with respect to the *S*_*t*_ became. The values of the spatial frequencies of *Co* and *St* were selected after running a first pilot experiment with seven healthy participants such that they were within the range used in literature (41, 42), considering the resolution of the robot (i.e., 0.02 mm), and stimuli that were not judged as too easy nor too difficult by the participants.

### 2.5. Haptic rendering of virtual textures

**Figure 3:**
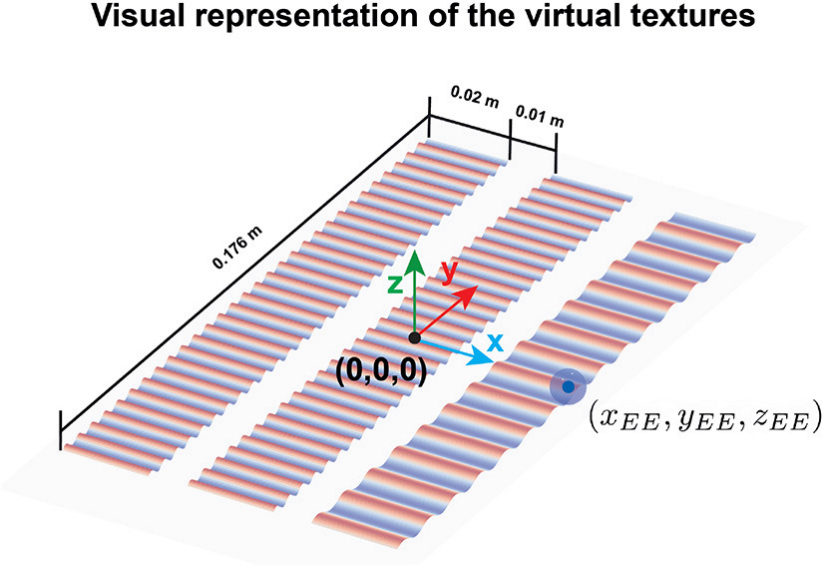
Visual representation of the three virtual textures. The coordinate center of the workspace is denoted by (0,0,0) and was located in the center of the second texture. The robot end effector is depicted with a blue ball whose center had the coordinates (*x*_*EE*_, *y*_*EE*_, *z*_*EE*_). The scale and size of the textures and the ball are only for visual purposes and do not represent the scale and size used in the experiment.

The virtual textures –visually represented in Figure 3—were rendered using the grating force calculated in equation 1 (see Section 2.4). The textures were rendered (i.e., through the force *F*_*rd*_) only on the *y*-direction and participants only perceived them when they were in contact with the texture, i.e., when the position of the robot end effector was below the virtual table height (*z*_*EE*_<*z*_*tbl*_ = 0.001*m*) and within the perimeter of the virtual textures in the *xy*-plane.


(2) $$F_{rd}=\left\{ \begin{array}{l} F_{g}\text{ if }contact\text{ is }True\;\\ \text{0 else.} \end{array}\right.$$

The three virtual textures laid on top of a haptic table of 0.20*m* x 0.10*m* whose surface was rendered by the robot using a Proportional-Derivative (PD) controller:


(3) $$F_{z}=\left\{ \begin{array}{l} K_{z}(z_{tbl}-z_{EE})+B_{z}(\dot{-z_{EE}})\text{ if }z_{EE}<z_{tbl}\\ 0\text{ else},\; \end{array}\right.$$

where the rendered force in the vertical direction *F*_*z*_ was proportional (*K*_*z*_ = 1960*N*/*m*) to the difference between the end effector vertical position *z*_*EE*_ and the height of the virtual table *z*_*tbl*_ = 0.001*m* when the robot end effector height was below the height of the virtual table (*z*_*EE*_<*z*_*tbl*_). The force in the vertical direction was zero otherwise. We added a damping element (*B*_*z*_ = 28*N*.*s*/*m*) to avoid excessive oscillations when the robot end effector was in contact with a rigid virtual surface (43). The robot was transparent in the *x*-direction at all times.

### 2.6. Exploration conditions: Active and passive touch

Participants explored the virtual textures under two different conditions: with active and passive touch. In this study, we employ the definition of passive touch provided by Symmons et al. (28), i.e., the act of touching an object while being assisted by an external agent (28). In our case, this external agent was the robot, which physically guided the participants' dominant hands during the exploration of the virtual textures. The haptic guidance *F*_*hg*_ was provided using the PD controller described in equation 4:


(4) $$F_{hg}=\left\{ \begin{array}{l} \overset{..}{y_{R}}+K_{hg}(y_{R}-y_{EE})+\text{ if }contact\text{ is }True\;\\ B_{hg}(\dot{y_{R}}-\dot{y_{EE}})\\ 0\text{ else,} \end{array}\right.$$

where *y*_*EE*_ was the *y* coordinate of the robot end effector position, i.e., the axis along the perceived textures, and $\overset{∙}{y_{EE}}$ its derivative. The stiffness *K*_*hg*_ was set to 300*N*/*m*, and damping *B*_*hg*_ to 60*N*.*s*/*m*. The reference trajectory –defined by $\ddot{y_{R}}$, $\overset{∙}{y_{R}}$, and *y*_*R*_– was obtained following the cycloidal motion law (described in Supplementary Section 1).

Participants were instructed to not oppose to the haptic guidance force and move along with the robot. They could move between textures by either exiting the textures sides in the *xy*-plane or by lifting the end effector (*z*-axis). Therefore, they were instructed not to lift the end effector while the guidance force was on.

Taking together equations 2, 3, and 4, the total force applied by the robot at the end effector was:


(5) $$\begin{array}{r} F_{Total}=F_{rd}+F_{z}+F_{hg}. \end{array}$$

### 2.7. Study protocol

**Figure 4:**
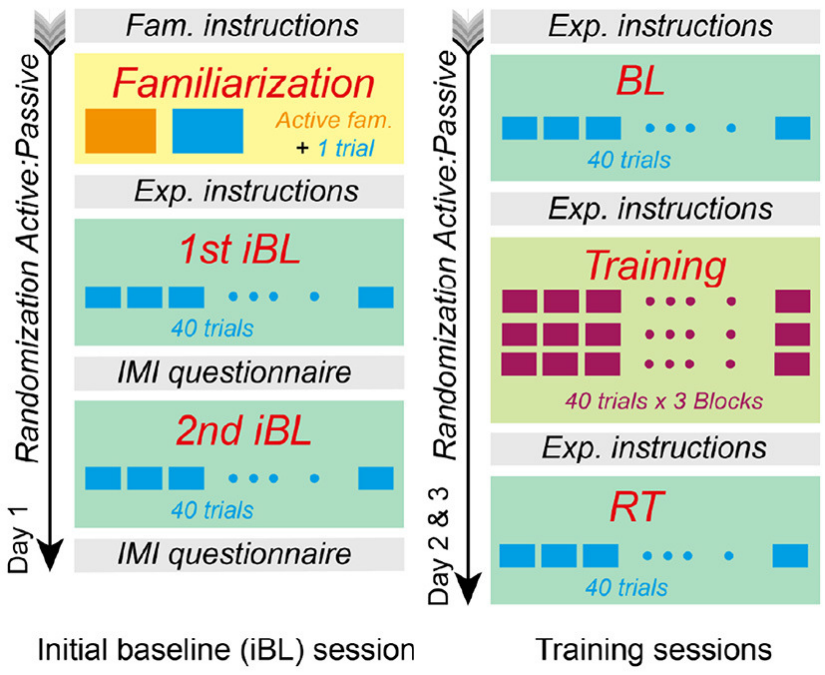
Experimental protocol. Participants completed three sessions on different days. The first session included familiarization and an initial baseline (iBL) for each condition. Half of the participants (randomly allocated) performed the first iBL in the active condition, whereas the second half had the passive condition first. We performed a second randomization to define the order of the training conditions during sessions 2 and 3. Each second and third session consisted of baseline (BL), training, and retention (RT) with either, passive or active conditions.

Figure 4 illustrates the experimental protocol of the within-subject experimental design. Participants completed three sessions, performing one session per day. There was a minimum of one to a maximum of 2 days between sessions.

#### 2.7.1. First session

**Figure 5:**
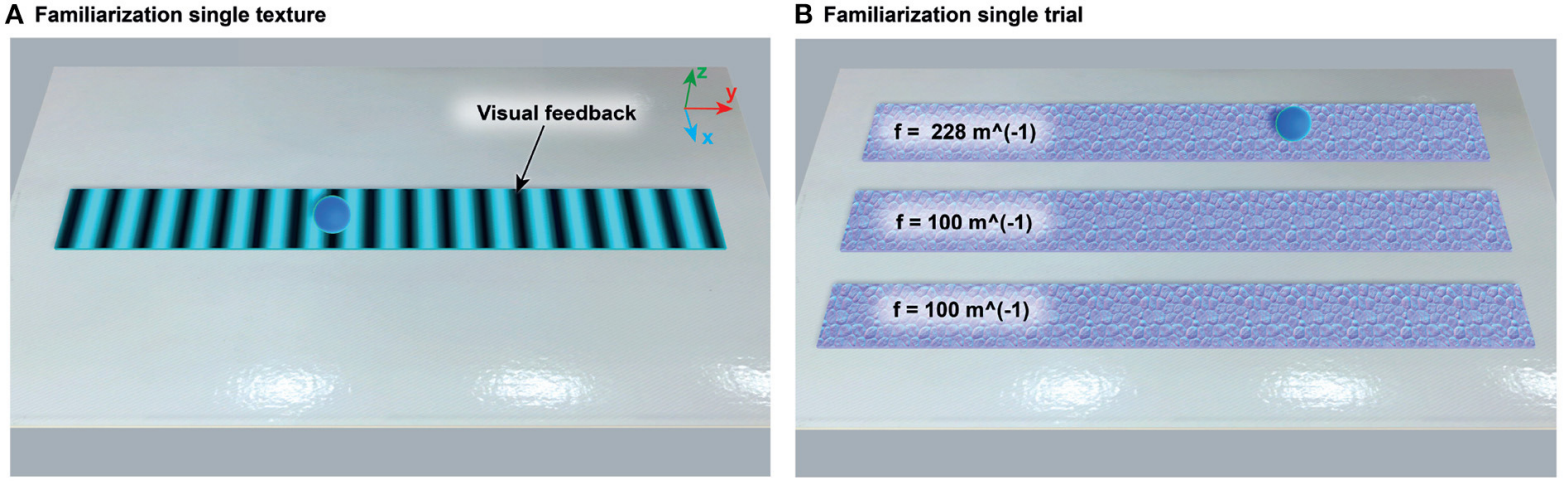
Virtual environments during familiarization. **(A)** The visual feedback that mapped the grating rendering was shown to help participants understand the haptic stimuli provided during the experiment. The dark color represented grooves, while the light blue color represented texture crests. Participants were encouraged to actively explore the texture as much as desired. **(B)** After the single texture familiarization, participants were asked to discriminate the odd texture among three virtual textures with identical visualization. The texture combination used for all participants was 228, 100, and 100 *m*^−1^, respectively.

The first session started with a **familiarization** phase followed by two initial baselines (iBL), which included one baseline per condition (i.e., active and passive touch). During the familiarization phase, all participants familiarized themselves with the robot and the experimental stimuli. Participants were invited to explore a single texture of 100 *m*^−1^, i.e., the more coarse texture in Table 2. During familiarization, we also provided visual feedback that mapped the haptic sensation (see Figure 5A) to facilitate the understanding of the virtual gratings. The dark color in Figure 5A represents the grooves, while the light blue color represents the texture crests. Subsequently, in a single familiarization trial, we asked them to select the odd texture among three textures that looked identical (see Figure 5B). The texture combination for all participants was 228, 228, and 100 *m*^−1^, respectively, from Table 2.

The first session included two **initial baseline** tests (iBL), one performed with the active and one with the passive condition. Half of the participants (randomly selected) performed the first iBL with the active condition and the second iBL with passive condition. The other half performed the iBLs in the contrary order. Each iBL included 40 trials, where each of the eight different comparison stimuli (*Co*) in Table 2 was presented a total of five times. The order of presentation of the stimuli was randomized for each participant and each condition.

Correct responses in each trial were registered following the criteria:


(6) $$Y_{i}=\left\{ \begin{array}{l} 1\text{ if the response is correct}\\ 0\text{ else,} \end{array}\right.$$

where *Y*_*i*_ represents the correctness of the response for each trial and stimuli *i*∈{1, 8}. The total number of correct responses was shown to the participant after finishing each iBL block. We saved the responses for all the 40 trials per iBL to compute the probability of correct responses (see Section 2.8).

#### 2.7.2. Sessions 2 and 3: Training

The sessions in the following experimental days included **baseline** (BL), **training**, and **retention** (RT) phases (Figure 4). Half of the participants (randomly allocated) performed the second session in the passive condition and the other half in the active condition. This was reversed in the third session, i.e., participants who performed the second session in the passive condition continued the third session with the active condition and vice versa. The BL and RT were consistent with the iBL session, i.e., the eight stimuli comparisons (*Co*) in Table 2 were presented a total of five times each. We randomized the stimuli presentation order for each phase (BL and RT).

The training phase consisted of 120 trials, grouped into three blocks of 40 trials each. Participants could rest for 2 min between training blocks. Each 40-trial block consisted of eight different comparison stimuli presented five times each. The comparison stimuli during training differed from those used in the baseline and retention phases (see Section 2.7.3). The stimuli presentation order was randomized for the first training block and repeated for the second and third training blocks.

**Figure 6:**
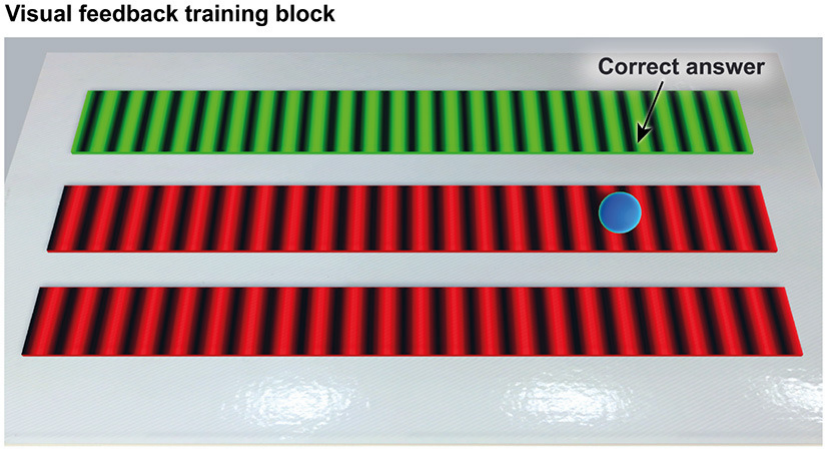
The terminal visual feedback provided after each trial during training. The green color represented the correct response (i.e., the odd texture), whereas the red color represented the twin textures (i.e., incorrect responses). The black lines were located along the grooves of the textures. Participants were allowed to re-explore the textures as desired.

During training, after each trial, we provided terminal visual feedback to the participants (Figure 6). This visual feedback consisted of 1) a texture turning green (i.e., the odd texture) and the two others red (i.e., incorrect responses), and 2) black parallel lines along the *x*-axis that represented the location of the texture grooves. Participants had then the opportunity to re-explore the textures as desired.

#### 2.7.3. Comparison stimuli during training

After completion of BL, we used a psychometric function to fit the probability of the participants having a correct response in BL based on the stimulus intensity –i.e., the absolute difference between the spatial frequency of the *Co* (*f*_*Co*_) and the spatial frequency of the *St* (*f*_*St*_), divided by the spatial frequency of the *St*). We employed the logistic function:


(7) $$\begin{array}{r} F\left(x|\alpha ,\beta \right)=\frac{1}{1+exp\left(-\left(\alpha +\beta x\right)\right)}, \end{array}$$

where α and β are the *intercept* and *slope* of the logistic function, respectively. Two logistic regressions were computed after the BL data were collected, one for the more coarse textures and a second one for the less coarse textures. Once the logistic functions were fitted to the BL data, we computed the point of subjective equality (PSE) by selecting the probability of a positive response π = 0.50 –i.e., the point at which two stimuli are perceived as one– for each participant and for each texture set, i.e., more and less coarse sets, *PSE*_*mc*_ and *PSE*_*lc*_, respectively. We then used these calculated values to create two new spanned ranges of comparison stimuli that were employed during training, i.e., we adapted the set of *Co* that were employed during training to each participants' performance during BL. Information about this process can be found in the Supplementary Section S2.

The fitting of the logistic functions did not always converge, i.e., the *PSE* was not within the range of the BL difference ratio [i.e., between 0.39 (–) and 0.098 (–)]. In those cases, the same *Co* stimuli used for BL and RT were employed during training.

### 2.8. Outcome variables

#### 2.8.1. Task performance

#### Probability of correct responses

The participants' texture discrimination performance was assessed using the probability of correct responses, calculated following the equation:


(8) $$\begin{array}{r} p_{i}=\frac{1}{n_{i}}\overset{n_{i}}{\underset{i=1}{\sum }}Y_{i}. \end{array}$$

The probability of correct responses was calculated for each comparison stimulus, denoted by the subindex *i*∈{1, 8}. The total number of times the response was correct was divided by the number of times *n*_*i*_ the *Co* stimulus was presented. For the iBL, BL, RT, and each training block of 40 trials, the total number of times each stimulus was presented was *n*_*i*_ = 5. The probability of correct responses for each stimulus was then averaged across all eight *Co* stimuli (four from more and four from less coarse textures) for each participant.

#### Point of subjective equality

The participants' psychometric task performance during BL and RT was also assessed using the point of subjective equality (PSE) (see Supplementary Equation S4). Compared to the probability of correct responses, the PSE is a performance metric that is more robust against participants' guessed responses. We averaged the PSE values for both the more coarse and less coarse textures. We only included PSE values that were within the range of the difference ratios provided during the experiment. The PSE values included were between 0.39 (–) and 0.098 (–). If a participant correctly responded to all trials, i.e., *p*_*i*_ = 1, the PSE score for that specific phase (i.e., BL or RT) was set to the minimum value of the difference ratio [i.e., 0.098 (–) during BL and RT]. The PSE could not be calculated when the logistic function did not converge. In those cases, we excluded from the data analysis paired cases in which PSE was not calculated either for BL or RT for a participant.

#### 2.8.2. Kinematic outcomes

We also evaluated the participants' texture exploratory behavior during BL and RT. In particular, per each trial, we calculated the scanning duration—i.e., the average time participants spent in contact with the three textures and moving faster than 0.01 m/s—, the path length—i.e., the path covered by the end effector over the texture averaged for the three textures—and the mean scanning speed—i.e., the mean end effector speed in *y*-direction.

#### 2.8.3. Motivation outcomes

We assessed the participants' subjective motivation after completing each active and passive iBL in session 1. Participants responded to 12 items selected from four subscales (i.e., Effort/Importance, Perceived Competence, Interest/Enjoyment, and Pressure/Tension) of the original Intrinsic Motivation Inventory (IMI) (44). Three items per subscale were included (see Supplementary Table S3 for the selected items). Participants rated each item using a 7-point Likert scale, with 1 indicating “not at all true” and 7 denoting “very true.” We averaged the answers of the three items for each subscale.

### 2.9. Data processing and statistical analysis

#### 2.9.1. System reliability

We estimated the system test-retest reliability—i.e., the correlation between two measurements from the same participant under the same conditions at distinct time points (45, 46)—by comparing the probability of correct responses and PSE scores between iBL (day 1) and BL (day 2) for each condition (passive and active touch).

We used the Intraclass Correlation Coefficient (ICC). The ICC reflects the degree of correlation and agreement between the participants' baseline (day 1 vs. day 2) measurements. The ICC value was estimated using the Python *pingouin*.*intraclass*_*corr* function and selecting the output of average random raters (47), i.e., considering an absolute agreement with multiple measurements. Reliability was considered excellent when *ICC* > 0.90, good when 0.75 < *ICC* ≤ 0.90, moderate when 0.5 < *ICC* ≤ 0.75 and poor otherwise (45).

To analyze the ICC for each condition, we allocated participants who performed the active condition on the second session to *ICC*_*active*_ group, whereas those who performed the passive condition on the second day were allocated to the *ICC*_*passive*_ group. Each group included 18 participants. We then compared the baseline in the second session BL of each condition group to their corresponding iBL in session 1 –note that on day 1, all participants performed an active iBL and a passive iBL. We did not consider BL on day 3 to avoid any training effects from the second day training. Absolute test-retest reliability was visually inspected using Bland-Altman plots for active and passive conditions for the probability of correct responses and the PSE.

#### 2.9.2. Training effects

For each task performance and kinematic outcome, we calculated the mean of the participants' BL and RT trials per condition—i.e., passive and active touch. The normal distribution of the outcome variables was verified using QQ-plots and the Shapiro-Wilk test.

To study the training effects on the task performance and kinematic outcome variables for each condition, we performed repeated-measures one-way ANOVAs—with factor *time*: BL and RL—for data with normal distribution. We analyzed non-normal data with Friedman tests.

We also evaluated the differences in training effects on touch sensibility between conditions –i.e., condition effects: Passive vs. Active– by comparing the pre-post changes (RT-BL) in task performance between active and passive conditions using repeated-measures one-way ANOVAs for data with normal distribution, and Friedman test for non-normal distributed data.

#### 2.9.3. Motivation

To analyze how active and passive conditions affected participants' motivation, we performed separated repeated measures one-way ANOVAs for each subscale –with factor *condition*: Active and Passive– with normal-distributed data, and Friedman test for non-normal distributed data.

The assumption of sphericity was met for all tests. We used the Python package “Pingouin” (48) for all the statistical tests. Finally, all statistical tests were set at a significance level of α = 0.05.

## 3. Results

### 3.1. System reliability

The ICC values for the probability of correct responses were computed for each condition. The active condition (*ICC*(2, *k*) = 0.497,  95% *CI* [−0.170, 0.800],  *p* = 0.055), and passive condition (*ICC*(2, *k*) = 0.518,  95% *CI* [−0.260, 0.820],  *p* = 0.069) showed poor and moderate reliability, respectively. Similarly, the ICC values for the PSE were computed for each condition. The active (*ICC*(2, *k*) = −1.885,  95% *CI* [−12.530, 0.200],  *p* = 0.955) had a poor reliability, whereas the passive condition (*ICC*(2, *k*) = 0.795,  95% *CI* [0.010, 0.950],  *p* = 0.025) showed a good reliability.

**Figure 7:**
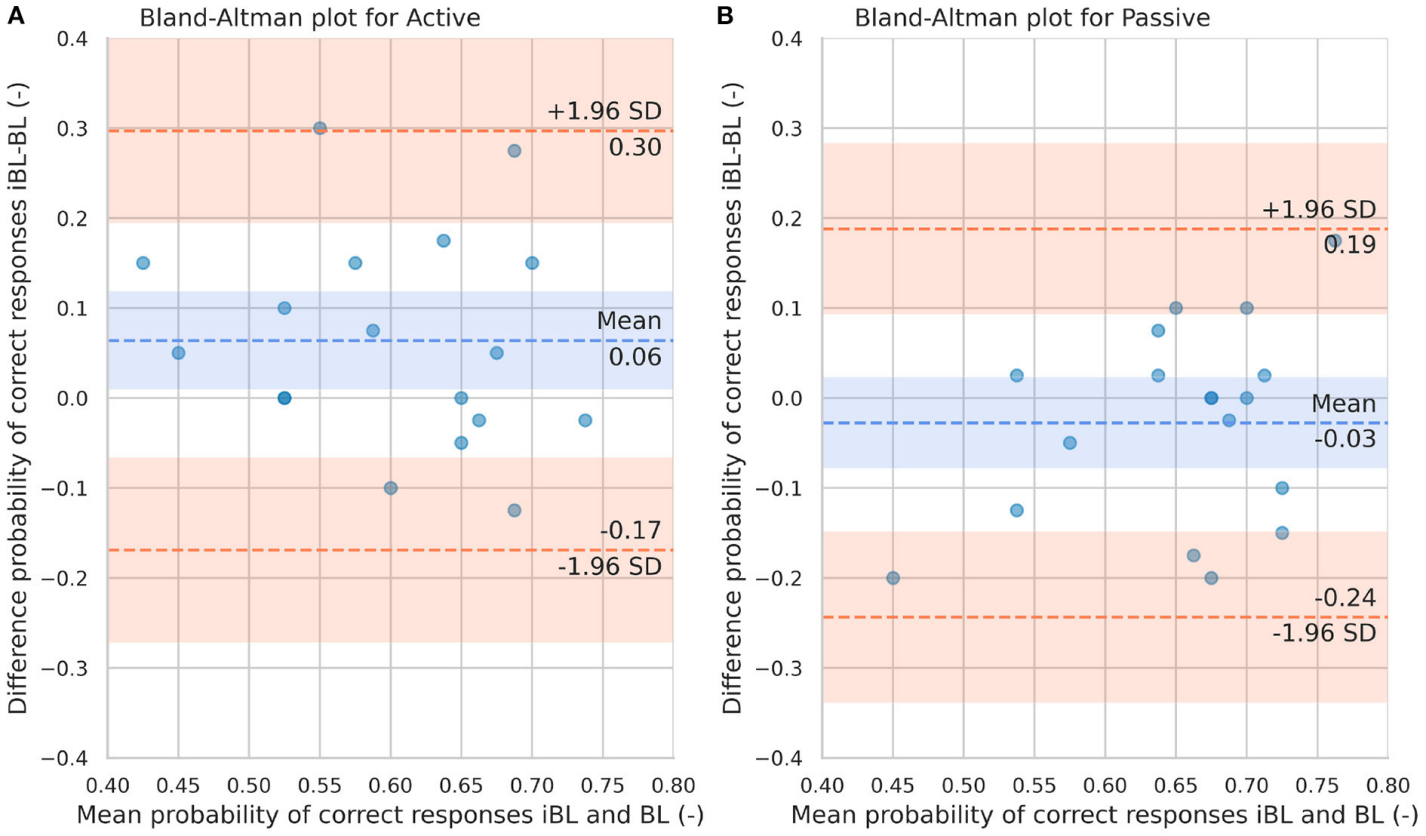
Bland-Altman plots for the probability of correct responses in iBL and BL for the Active condition **(A)** and Passive condition **(B)**. The blue dashed line represents the mean difference between iBL and BL. The lower and upper orange dashed lines represent the lower and upper 95% confidence limits, respectively.

**Figure 8:**
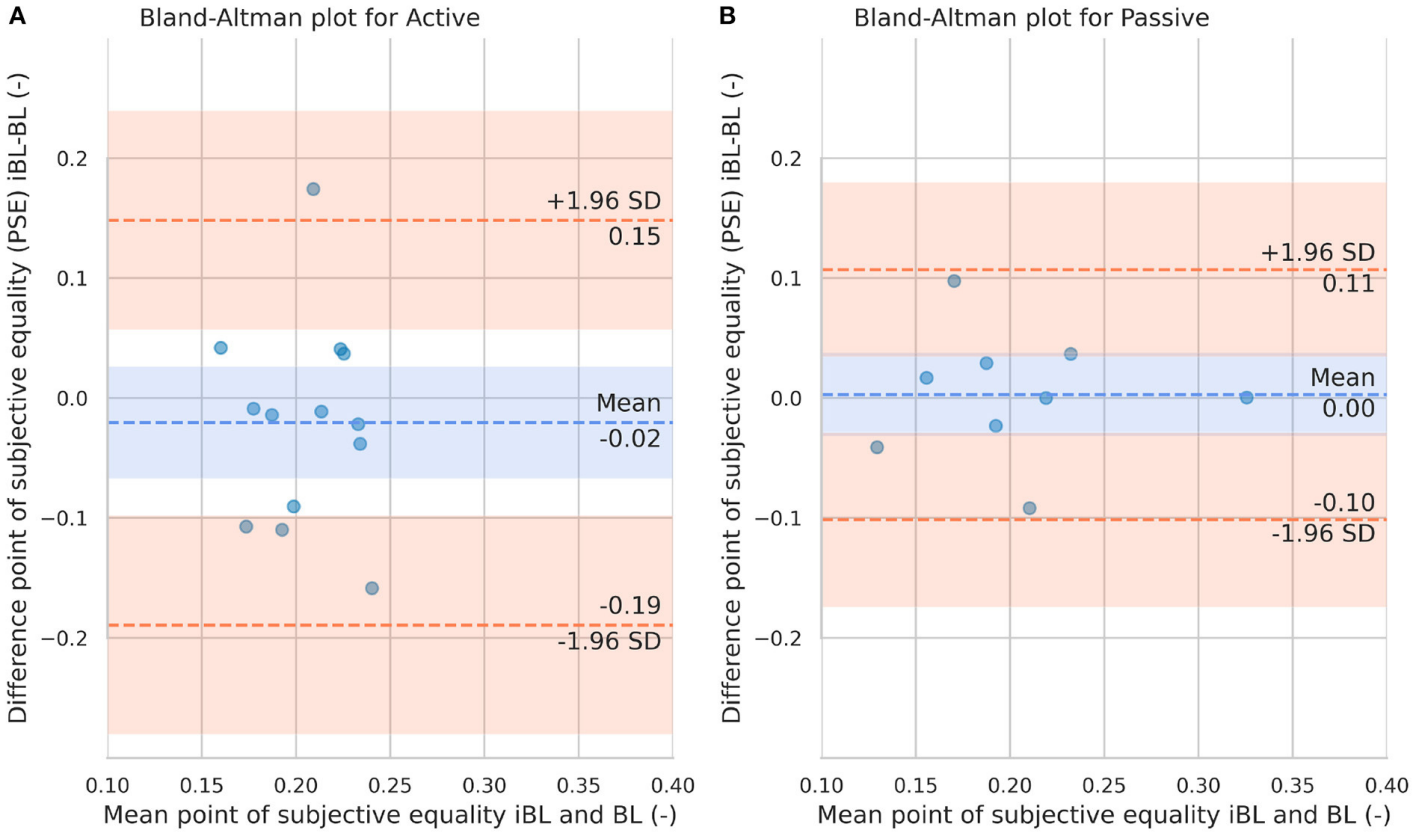
Bland-Altman plots for the point of subjective equality in iBL and BL for the Active condition **(A)** and passive condition **(B)**. The blue dashed line represents the mean difference between iBL and BL. The lower and upper orange dashed lines represent the lower and upper 95% confidence limits, respectively.

The Bland-Altman plots for the probability of correct responses and PSE for active and passive conditions are shown in Figures 7, 8, respectively. From Figure 7A it can be observed that in just one participant the difference between iBL and BL measurements for the probability of correct responses is over the upper bound of the 95% CI, while the mean of the differences between baselines is around 0.06 (-). From Figure 7B, it can be seen that the difference between iBL and BL is closer to zero [–0.03 (-)] compared to the active condition. Figure 8A shows that there was a participant whose PSE difference between iBL and BL for the active condition was outside of the CI. On the contrary, all data points in Figure 8B for the PSE in passive condition were within the 95% CI with zero mean.

### 3.2. Task performance

**Figure 9:**
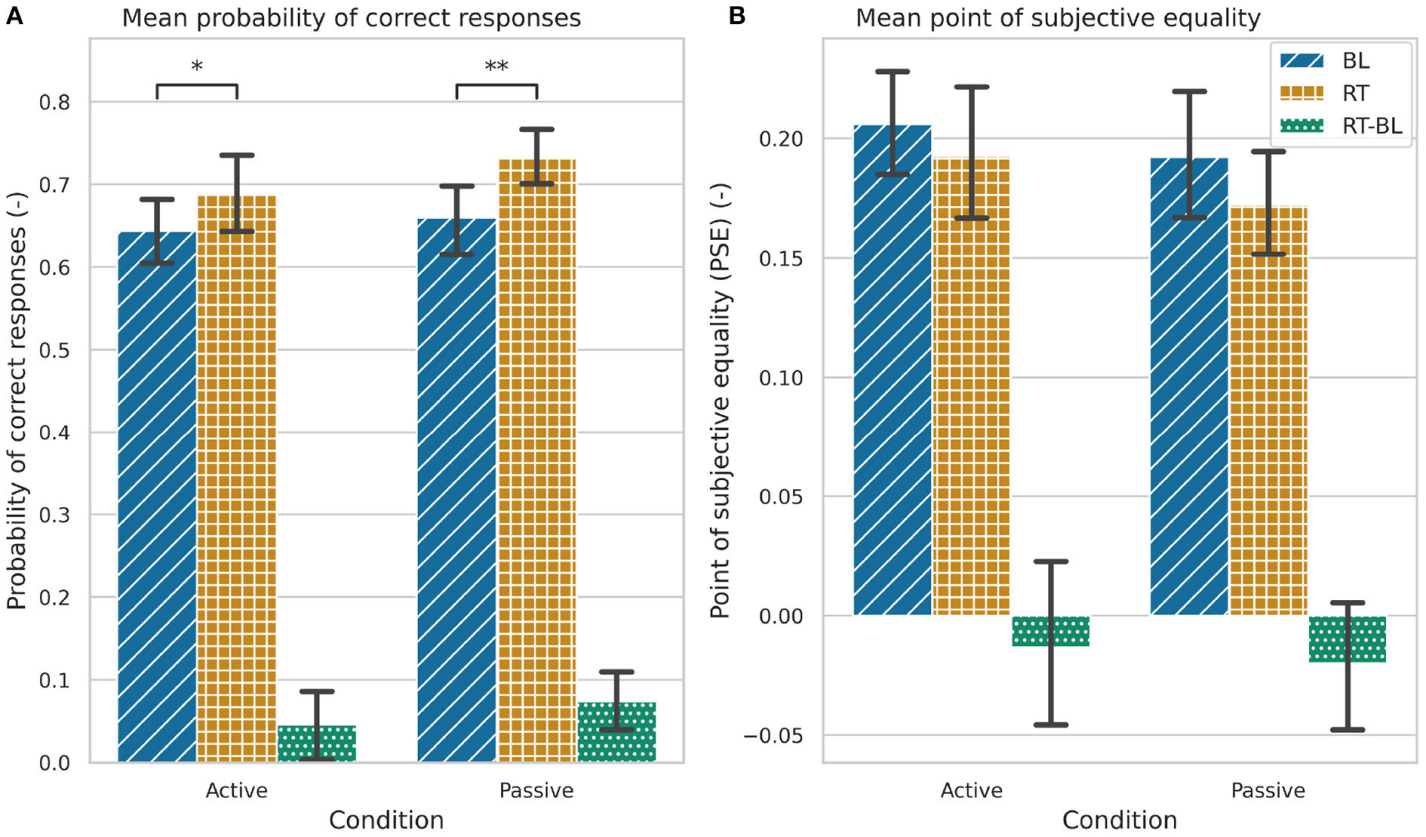
Task performance during baseline (BL), retention (RT), and the changes between baseline and retention (RT-BL) for the active and passive conditions. **(A)** Probability of correct responses. **(B)** Point of subjective equality (PSE). Statistically significant differences are marked by (*p < 0.05), (**p < 0.001). The error bars represent the confidence intervals.

Participants significantly improved the probability of correct responses from BL to RT in the active [$X_{\left(1,35\right)}^{2}=4.235$, *p* = 0.039; Figure 9A and Table 3] and passive conditions [*F*_(1, 35)_ = 15.564, *p* < 0.001; Figure 9A and Table 3]. We found no significant differences between the active and passive conditions in the pre-post training changes in the probability of correct responses (Table 4).

**Table 3:** Overview of results for task performance and kinematic outcome variables in active and passive conditions during the baseline (BL) and retention (RT) phases.

	**Training effects**					
**Independent variables**	**Active**			**Passive**		
	**BL**	**RT**	***p*-value**	**BL**	**RT**	***p*-value**
Task performance						
Probability of correct responses (-)	0.644 (0.120)	0.725 (0.116)	**0.039**	0.660 (0.124)	0.733 (0.105)	**<** **0.001**
Point of subjective equality (-)	0.206 (0.058)	0.193 (0.073)	0.450	0.192 (0.068)	0.172 (0.053)	0.163
Kinematic outcomes						
Scanning duration (s)	4.418 (1.537)	4.125 (1.624)	0.153	3.969 (1.569)	3.954 (1.514)	0.931
Path length (m)	0.804 (0.281)	0.842 (0.338)	0.356	0.820 (0.259)	0.788 (0.221)	0.343
Scanning speed (m/s)	0.175 (0.043)	0.194 (0.053)	**0.001**	0.172 (0.009)	0.176 (0.007)	**<** **0.001**

*Mean and standard deviation (in brackets) are reported when the data were normally distributed and median and mean absolute deviation (in brackets) when the data were non-normal. Statistically significant values are highlighted in bold*.

**Table 4:** Overview of results comparing the pre-post changes from baseline (BL) to retention (RT) in active vs. passive conditions.

	**Active vs. Passive**		
**Independent variables**	**Active**	**Passive**	
	**RT-BL**	**RT-BL**	***p*-value**
Task performance			
Probability of correct responses (-)	0.046 (0.124)	0.074 (0.112)	0.323
Point of subjective equality (-)	0.013 (0.087)	0.020 (0.063)	0.285
Kinematic outcomes			
Scanning duration (s)	–0.293 (1.203)	–0.015 (1.028)	0.261
Path length (m)	0.039 (0.248)	–0.033 (0.206)	0.151
Scanning speed (m/s)	0.020 (0.034)	0.004 (0.006)	**0.008**

*Mean and standard deviation (in brackets) are reported when the data were normally distributed and median and mean absolute deviation (in brackets) when the data were non-normally distributed. Statistically significant values are highlighted in bold*.

Participants did not significantly improve their PSE values from BL to RT in any of the conditions (Figure 9B and Table 3). There were also no significant differences in the RT-BL changes between the conditions (Table 4).

### 3.3. Kinematic outcomes

**Figure 10:**
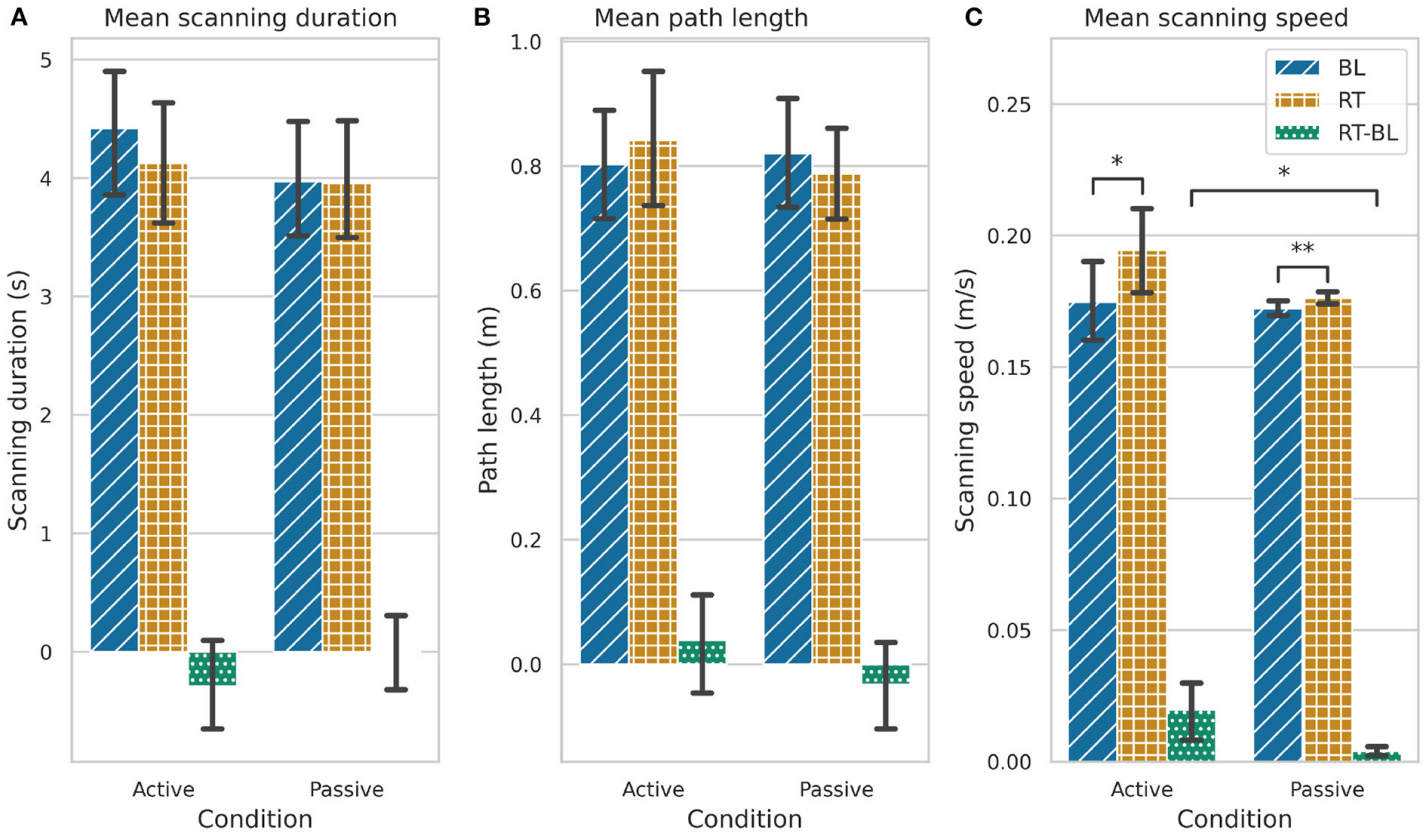
Kinematic outcomes during baseline (BL), retention (RT), and the changes between baseline and retention (RT-BL) for the active and passive conditions. **(A)** Scanning duration. **(B)** Path length. **(C)** Scanning speed. Statistically significant differences are marked by *p < 0.05, **p < 0.001. The error bars represent the confidence intervals.

We did not find significant changes in the scanning duration or path length from BL to RT in any of the conditions, neither we found differences in the RT-BL changes between conditions (Figures 10A,B and Tables 3, 4). However, we found that participants performed faster exploratory movements after training with both conditions [active: *F*_(1, 35)_ = 12.121, *p* = 0.001; passive: *F*_(1, 35)_ = 17.989, *p* < 0.001; Figure 10C and Table 3]. The increase in scanning speed was significantly higher in the active condition compared to the passive condition [*F*_(1, 35)_ = 8.017, *p* = 0.008, Figure 10C and Table 4].

### 3.4. Motivation

We did not find significant differences between active and passive conditions in the first experimental session in the IMI subscales Effort/Importance, Interest/Enjoyment, and Pressure/Tension (Table 5). However, we found a significant difference between active and passive conditions in the Perceived Competence subscale [*F*_(1, 35)_ = 9.701, *p* = 0.004, Table 5].

**Table 5:** The results for each motivation subscale comparing the differences between initial baseline (iBL) active and initial baseline passive.

	**Active vs. Passive**		
**Independent variables**	**Active**	**Passive**	
	**iBL**	**iBL**	***p*-value**
**Motivation**			
Effort/Importance (-)	5.213 (1.130)	5.028 (1.018)	0.108
Perceived Competence (-)	2.833 (1.085)	3.296 (1.041)	**0.004**
Interest/Enjoyment (-)	4.574 (1.407)	4.694 (1.470)	0.245
Pressure/Tension (-)	2.000 (1.061)	2.000 (0.877)	0.368

*Mean and standard deviation (in brackets) are reported when the data were normally distributed and median and mean absolute deviation (in brackets) when the data were non-normal. Statistically significant values are highlighted in bold*.

## 4. Discussion

We developed and evaluated a novel virtual reality-based haptic system to assess and train touch discrimination. The novelty of our system is that the haptic rendering forces of the textures are applied tangentially to the hand and do not depend on the normal force that participants exert on the texture surface, as is the case when using real textures. Thus, by disentangling the tangential and normal forces, we aimed to provide more controlled stimuli within and between participants.

Thirty-six healthy participants were asked to discriminate virtual textures using active and passive conditions, i.e., with the robot not guiding their movements vs. the robot guiding their hands. We evaluated the reliability of our system and the potential to train tactile sensibility in a within-subjects three-session experiment.

### 4.1. The system reliability

We evaluated our system reliability by comparing the baseline tests on two different days. We found that the reliability with respect to the probability of correct responses ranged from poor to moderate in active and passive conditions, respectively. The reliability relative to the PSE differed between conditions; although our system showed good reliability in the passive condition, the reliability in the active condition was rather poor. This is in line with our hypothesis of better reliability in the passive condition as by guiding the movements with the robot, we provided a more controlled texture exploration.

It is recommended that the test-retest ICC value should be at least 0.90 if the system is aimed to assess or evaluate the treatment of a patient (46). Our system did not reach excellent reliability in any of the performance metrics evaluated with 36 healthy participants (18 subjects per condition). Yet, several studies have shown poor to moderate reliability for sensory assessment in a healthy population, e.g., when using robotic devices (49), and physical textures (50). It has also been observed lower reliability values in robotic sensory assessments in the unimpaired limb compared to the impaired limb in stroke patients (49). This has been suggested to be due to the dependency of the ICC on the between subject variability, that in a healthy population is usually small. Thus, we expect that including brain-injured patients in future studies would result in higher reliability values, especially when testing the impaired limb—e.g., excellent reliability in a tactile discrimination test that required stroke patients to discriminate differences in physical finely graded ridged surfaces was observed in an experiment with 35 patients (51).

There may be other reasons for the limited reliability observed in our system. First, contrary to Carey et al. (51), we did not account for a possible lack of familiarization in the first session. Although we did add a short familiarization phase, it only included one trial. Thus, probably between the two sessions, learning occurred. Furthermore, the familiarization was only performed with the active condition. Second, in our 3-day experiment, participants experienced both conditions in the first session. Some participants, therefore, first had an active and then passive baseline condition on day 1, followed by an active condition on day 2, while some participants had an active and then passive baseline on the first day, followed by a passive condition on the second day. Thus, in some cases, there was a baseline with the other condition between two baselines of the same type (iBL and BL) that could have served as “training,” potentially hampering the reliability. Thus, our results support the idea that more extensive familiarization trials should be performed prior to clinical assessments. Finally, the variance of the outcome measurements between sessions may be a result from the spontaneous fluctuations (i.e., noise) of the somatosensory system (52).

The ICC has been shown to be sensitive to intra- and inter-subject variability and that reporting the CI for reliability is also important (53). Yet, reporting ICC values together with the CI alone might provide insufficient information for a reliability analysis (54). Therefore, to extend our system reliability analysis and visually inspect the agreement between the first (iBL) and second measurements (BL), we performed Bland-Altman plots for both performance metrics. Bland-Altman plots on the proportion of correct responses and PSE can be employed to observe the magnitude of measurement error of each test-retest difference (iBL-BL). In the case of the passive condition, the difference between the two measurements was relative low and all the data points laid within the limit of confidence (chosen as 95%) in both performance metrics. However, in the active condition some data points laid outside the CI boundaries in both performance metrics. Thus, using the robot to guide the movements during passive texture exploration seems a more reliable assessment tool than allowing participants to freely explore. This finding will guide next experiments with brain-injured patients. It seems reasonable to not include active touch during follow-up experiments with patients. This will reduce the duration of future experiments, leveraging more promising techniques while preventing patients from engaging in too exhausting interventions.

### 4.2. Passive and active robotic somatosensory training enhances the discrimination of virtual textures

Consistent with our hypothesis, we found an improvement in virtual texture discrimination after training with both active and passive conditions. Participants significantly improved their tactile acuity of virtual textures, as reflected in a significant increase in the probability of correct responses from baseline to retention after three training blocks of active or passive conditions. Although we also observed a decrease in the PSE after training with active and passive conditions, the difference did not reach significance. This might be due to the lower number of sample points included in the PSE statistical analysis, compared to the larger sample in the probability of correct responses analysis. Several BL and RT trials (active: 22 out of 72, and passive: 30 out of 72) had to be removed from the statistical analysis as the fitting of the logistic function did not converge.

Our results are in line with several studies that evaluated the potential of sensory retraining strategies, e.g., Elangovan et al. (15), Ballardini et al. (23), and Carey et al. (2). Most studies to date focused on training proprioception rather than tactile perception. For example, Elangovan et al. (15) and Yeh et al. (16) found enhanced proprioceptive function after an active proprioception retraining intervention that required healthy participants (15) and stroke patients (16) to balance a virtual ball on a virtual table. Improvements in tactile perception were also found in a few studies that evaluated tactile training interventions in healthy [e.g., Ballardini et al. (23)] and stroke patients [e.g., Carey et al. (2)]. Ballardini et al. (23) found enhanced tactile sensitivity after a sensory retraining task that required healthy participants to discriminate and replicate skin-brushed stimuli that were applied by the end effector of a robotic device on the palm of their dominant hand. In each training trial, participants were asked to actively move their non-dominant hands and reproduce the stimulus that they previously experienced at several target locations on the palm of their dominant hands. Further, Carey et al. (2) found clinically and statistically significant improvements in the ability to discriminate differences in tactile stimuli after 13–16 weeks of training. Carey et al. (2) assessed and trained touch sensibility using a set of fine plastic gratings, which differed only by their spatial periods. Textures were presented in sets of three, with and odd texture among two identical ones, and –as in with our experiment– patients were asked to select the odd texture. However, unlike our work, Carey et al. (2) did not adjust the difficulty of the training based on the specific baseline performance of the patients. As a novelty, our sensory retraining strategy followed an adaptive intervention that adjusted the difficulty of the task to meet the specific needs of the participants. However, we did not compare differences on the effect of training with adaptive vs. fixed difficulty levels on tactile discrimination, and therefore, conclusions about the suitability of our adaptive training program cannot be driven.

Carey et al. (2) and Sathian and Zangaladze (55) reported that training effects appear to be stimulus-specific and task-specific. Therefore, improvements in texture discrimination due to our intervention may not be transferable to other types of tactile perception tasks –e.g., recognition of haptic letters (33). Further, we cannot rule out that the performance improvements observed after our tactile discrimination training may result from a better understanding of the task rather than reflect improvements in individual touch sensibility. The addition of visual feedback after each training trial could have helped participants to better understand the task and, consequently, improve their performance. However, it should be noted that most of the participants trained with spatial frequencies that were different from those presented during baseline and retention as we adapted the training comparison stimuli based on the participants' individual PSE at baseline. More precisely, for the active training sessions, the logistic regression for the less coarse textures converged in 25 of the 36 participants (and therefore assembling a new set of training comparison stimuli), and for the more coarse textures in 23 of 36 participants. For the passive training sessions, the logistic regression converged in 24 of 36 participants for both less and more coarse textures. The fact that participants trained with stimuli different from those presented at baseline and retention minimizes the possibility that our findings reflect stimulus-specific training effects.

Although we found significant differences in the participants' discrimination performance after training, these did not seem to be related to pre-post training changes in the textures exploration behavior. We did not find significant changes in the scanning duration nor the path length on the textures after training. However, we did find a small, albeit significant, increase in the scanning speed after training for both active and passive conditions. Yet, several researchers showed that tactile perception is invariant to changes in exploration speed (56–58).

### 4.3. No differences between active and passive conditions in training effects on touch sensibility

As hypothesized, we did not find differences in the effect of active vs. passive conditions on pre-post changes regarding the probability of correct responses nor PSE values. Our results are consistent with previous findings, which revealed no differences between active and passive conditions (33, 56, 59). Lederman (59) found no differences between active and passive conditions when their participants estimated magnitudes of the roughness of metal gratings (59). Vega-Bermudez et al. (33) found no differences in tactile recognition of letters between active and passive conditions. Further, Lamb (56) found no differences between active and passive conditions in discriminating between plastic strips with raised dots. However, contrary to our experiment, in all these studies (33, 56, 59) participants received the passive stimulation with their arms and hands immobilized, e.g., by using a drum stimulation. Thus, the active conditions did not provide an advantage over the passive condition due to the addition of, for example, kinesthetic cues associated with the active movement.

Vega-Bermudez et al. (33) reported that the majority of experiments that found differences between active and passive conditions employed tasks in which proprioceptive information represented a critical component for the success of the sensory task. In our study, we compared the active condition to a passive condition in which the robot guided the participants' hands and thus also provided proprioceptive information during the tactile discrimination task. Our findings, therefore, extend previous studies by suggesting that there are no differences between active and passive touch even when controlling for kinesthetic information between conditions.

A potential rationale behind the lack of differences between passive and active conditions is that in both conditions participants explored the textures using indirect contact, i.e., through the robot end-effector. In the haptics field, tactile texture perception has been investigated in two modes of touch contact: direct and indirect contact. Direct contact refers to participants touching (or being touched by) an object with (in) their bare skin –e.g., their fingertips–, whereas indirect contact refers to touching objects using an intermediary link –e.g., gloves, tools, or robotic devices (60, 61). During direct touch, the roughness of the texture is spatially coded by the central nervous system using tactile information sensed by mechanoreceptors on the glabrous skin. Temporal cues are also temporally coded using vibration cues (62). The weight of the relative use of spatial and temporal cues seems to vary depending on the spatial period of the texture (61). However, when people explore a texture indirectly through a tool, the roughness perception of textures seems to be mainly coded *via* temporal cues (61). During indirect touch, the spatial information of the texture is usually no longer available as the spatial deformation of the skin relates to the shape of the probe rather than the properties of the scanned texture (63). Consequently, in our indirect touch experiment, participants received similar vibratory cues to perform the sensory task under both conditions, which may explain the lack of significant differences between the active and passive touch conditions.

### 4.4. Participants' motivation

We hypothesized that haptic guidance during training would improve participants' perceived competence. As expected, we found that participants reported significantly higher competence during passive than active exploration, which may be a positive indicator for using passive exploration with brain-injured patients. The robotic guidance during training might allow participants to focus on the sensory input, without the need to think about how to explore the textures (instead, the robot takes the “responsibility”), therefore, increasing their subjectively reported perceived competence. Physically and cognitively impaired patients could likely even further benefit from this guidance, as it allows them to focus on the task instead of the exploration strategy, potentially increasing the effects of sensory training.

We also hypothesized that during active exploration, participants would report higher levels in effort, pressure, and interest/enjoyment than during passive exploration, as exploring the textures themselves may make the training more engaging, but may also be associated with the challenge to choose an optimal exploration strategy. Contrary to our hypothesis, we found no differences between active and passive conditions on the IMI subscales Effort/Importance, Interest/Enjoyment, and Pressure/Tension. This result may indicate that participants remained engaged in the task under both conditions, even though they did not need to actively move along the textures during training with the passive condition. However, overall, we found high Effort/Importance reportings, whereas the perceived competence was quite low, indicating that the experiment was rather challenging. Hence, in future experiments and especially with brain-injured patients, it is important to lower the difficulty of the task, e.g., by reducing the number of stimulus comparisons or augmenting the interstimulus distance to simplify the task. Additionally, attention may influence the perception of virtual textures.

### 4.5. Study limitations and future work

Our study suffers from several limitations. First, participants were allowed to freely explore the textures, without any time limitation. We decided to allow for “free” exploration while measuring the kinematic data –i.e., scanning duration, end effector path length, and scanning speed– during exploration to evaluate the effect of training with the different conditions on the exploration strategies after training. Second, the long duration of the training blocks might have triggered the so-called paresthesia, i.e., the sensation experienced as a numbness or tingling sensation on the skin (12) and a result of excessive sensory stimulation without long enough resting periods. Yet, we still found improvements in the discrimination of the virtual textures right after the training finished (in short-term retention). Third, contrary to Carey et al. (2) and Ballardini et al. (23), we did not blindfold the participants, i.e., we did not occlude the vision of the hands and/or robot. Instead, we provided visual feedback using a virtual environment to motivate participants. Yet, the use of the virtual environment to provide visual feedback on the participant's hand position with respect to the virtual textures, which was located in a different space than the robot/hand movements, probably prevented participants to look at their own hands as they were focused on the screen visualization.

Although our system showed moderate to good reliability values with 18 healthy participants per condition, the reliability evaluation would benefit from including more healthy participants, and especially brain-injured patients. It has been observed lower reliability values in healthy compared to stroke patients (49, 50), and thus, we expect to observe higher reliability values when assessing texture discrimination in a brain-injured population. Moreover, in future experiments, we plan to test the feasibility and acceptability of our system with brain-injured populations, as the majority of robotic devices in neurorehabilitation is tested with healthy populations instead of patients. Yet, several changes must be performed to our protocol before bringing our system to the clinics. First, we suggest performing the experiment with brain-injured patients only with the passive condition, as our results with healthy participants suggest that training effects would not differ between conditions and the passive condition shows higher reliability. Second, the training duration should be reduced to prevent paresthesia, e.g., by reducing the number of trials and repetitions per stimuli. Third, we may need to adapt the location of the monitor to account for patients with visual neglect, as it was found that visual neglect might interfere with the assessment of somatosensory impairment probably due to attention deficit (64). Further, we may need to adjust the level of discrimination difficulty accordingly to the patients' deficits. Finally, to increase the system reliability, we suggest increasing the familiarization time, controlling the time between sessions (with a minimum of 24 h between sessions) and the time of the day the intervention is delivered.

## 5. Conclusion

Despite the high prevalence of sensory deficits after stroke, somatosensory treatment is currently neglected in neurorehabilitation interventions. Crucially, there is a lack of high-quality research demonstrating benefits of somatosensory (re)training on stroke recovery and a need for reliable quantitative assessments of sensorimotor deficits. Further, to date, somatosensory assessments and interventions are labor-intensive and require therapists to guide the paretic limbs of the patients. The goal of this study was to develop and evaluate the reliability of a novel virtual reality-based robotic texture discrimination task that allows to assess and train touch sensibility with (i.e., passively) and without (i.e., actively) guidance for potential clinical application.

In our sample of healthy young participants with expected low between-subject variability, our system showed poor (in active condition) to moderate/good (in passive condition) reliability. Furthermore, we found that participants significantly improved their task performance after training and that these training effects did not differentiate between active and robotic-guided passive exploration. Similarly, both conditions did not differ in motivation, except that passive touch sensibility training was associated with increased perceived competence.

Together, our virtual reality-based robotic haptic system may be a key asset for the evaluation and retraining of sensory loss with minimal supervision, especially for brain-injured patients who require guidance to move their limbs.

## Data availability statement

The datasets of this study are available in the following repository: http://doi.org/10.5281/zenodo.6434872. Further details can be directed to the corresponding author/s.

## Ethics statement

The studies involving human participants were reviewed and approved by Swiss Cantonal Ethics Committee (Basec ref: 2018-01179) and the Swiss Agency for Therapeutic Products (Swissmedic ref: 100000432). The participants provided their written informed consent to participate in this study. Written informed consent was obtained from the individual(s) for the publication of any potentially identifiable images or data included in this article.

## Author contributions

EV, EA, KB, and LM-C designed the study and wrote the manuscript. EV performed the analysis of the dataset, prepared the experimental setup and programmed the virtual environment and robot, and collected the experimental data. All authors edited and revised the manuscript and approved the submitted version.

## Funding

This work was supported by the Swiss National Science Foundation through the grant PP00P2 163800. EV was sponsored by SENACYT-IFARHU with the Doctoral and postdoctoral fellowship grants subprogram.

## Conflict of interest

The authors declare that the research was conducted in the absence of any commercial or financial relationships that could be construed as a potential conflict of interest.

## Publisher's note

All claims expressed in this article are solely those of the authors and do not necessarily represent those of their affiliated organizations, or those of the publisher, the editors and the reviewers. Any product that may be evaluated in this article, or claim that may be made by its manufacturer, is not guaranteed or endorsed by the publisher.
