# Supplementary material for: Enhancing touch sensibility by sensory retraining in a sensory discrimination task via haptic rendering
Source: Front Rehabil Sci. 2022 Aug 1;3:929431. doi: 10.3389/fresc.2022.929431 (PMC9397824; doi:10.3389/fresc.2022.929431)
Supplement: Supplementary file 1 [file Data_Sheet_1.PDF]

Supplementary Material

1 THE CYCLOIDAL MOTION LAW

The reference trajectory –defined by $\ddot{y_R}$, $\dot{y_R}$, and y_R– was obtained following the cycloidal motion law (described in Biagiotti and Melchiorri (2008), page 44):

$$y_R(t) = y_{R0} + dS\left(\frac{t}{T} - \frac{\sin\left(2\pi\frac{t}{T}\right)}{2\pi}\right), \tag{S1}$$

from which

$$\begin{aligned} \dot{y_R}(t) &= \frac{dS}{T}\left(1 - \cos\left(2\pi\frac{t}{T}\right)\right) \\ \ddot{y_R}(t) &= \frac{2\pi dS}{T^2}\left(\sin\left(2\pi\frac{t}{T}\right)\right), \end{aligned} \tag{S2}$$

where y_{R0} is the y-position of the end effector when the participant entered or landed into the texture. Participants could enter the texture from any of the four sides of each rectangular texture or lift the robot end effector and land on top of the texture. The displacement dS was calculated each time the participant entered the texture as the maximum distance between y_{R0} and the farthest short-edge of the texture (Fig. 3 in the main manuscript). Thus, participants were guided in the y-direction towards the end of the texture that was farthest away from the initial position. The t represented the internal clock of the robot controller with a sampling rate of 4 kHz and T was set to 1 second (i.e., the time needed to finish the movement). By using the cycloidal motion law we could ensure a smooth trajectory reference each time a participant entered a texture.

2 ADJUSTED COMPARISON STIMULI FOR TRAINING

Once the logistic functions were fitted to the BL data, we computed the point of subjective equality (PSE) by selecting the probability of a positive response π = 0.50 –i.e., the point at which two stimuli are perceived as one– for each participant and for each texture set, i.e., more and less coarse sets, PSE_{mc} and PSE_{lc}, respectively). The PSEs were calculated from the inverse of the logistic function as:

$$x = F^{-1}(\pi|\alpha, \beta) = \frac{1}{\beta}\left(\log\frac{\pi}{1-\pi} - \alpha\right) \tag{S3}$$

$$PSE = F^{-1}(0.5|\alpha, \beta) = \frac{1}{\beta}\left(\log\frac{0.5}{0.5} - \alpha\right) = -\frac{\alpha}{\beta}. \tag{S4}$$

We used the PSE_{mc} and PSE_{lc} calculated for each participant to create two new spanned ranges of comparison stimuli that were employed during training, i.e., we adapted the set of Co that were employed during training to each participants' performance during BL.

We used the mathematical expressions in equations S5 to S8 to set the new frequency difference between consecutive comparison stimuli (fixed to 16 m^{-1} during BL and RT) for the more coarse textures f_{mc} and less coarse textures f_{lc}.

$$fpse_{mc} = |f_{St} - PSE_{mc} \cdot f_{St}| \tag{S5}$$

$$fpse_{lc} = |f_{St} + PSE_{lc} \cdot f_{St}| \tag{S6}$$

$$f_{mc} = \frac{|f_{St} - fpse_{mc}|}{n} \tag{S7}$$

$$f_{lc} = \frac{|f_{St} - fpse_{lc}|}{n}, \tag{S8}$$

where n is the number of new comparison stimuli employed during training per coarse type, that was fixed again to $n = 4$. Thus, the new set of comparison stimuli for the more coarse textures was set to:

$$f_{Co_{more\ coarse}} = [f_{St} - f_{mc}, f_{St} - 2f_{mc}, f_{St} - 3f_{mc}, f_{St} - 4f_{mc}] \tag{S9}$$

while the new set of comparison stimuli for the less coarse textures was set to:

$$f_{Co_{less\ coarse}} = [f_{St} + f_{mc}, f_{St} + 2f_{mc}, f_{St} + 3f_{mc}, f_{St} + 4f_{mc}]. \tag{S10}$$

3 MOTIVATION QUESTIONNAIRE

Table S1. Subscales and items selected from the "Intrinsic Motivation Questionnaire" (Ryan et al., 1990).

Subscale	Questions
Effort/Importance	I tried very hard on this activity.
	I put a lot of effort into this.
	It was important to me to do well at this task.
Perceived Competence	I am satisfied with my performance at this task.
	I think I am pretty good at this activity.
	I was pretty skilled at this activity.
Interest/Enjoyment	The task was fun to do.
	I thought this activity was quite enjoyable.
	I would describe this activity as very interesting.
Pressure/Tension	I felt very tense while doing this activity.
	I felt pressured while doing these.
	I was anxious while working on this task.

REFERENCES

Biagiotti, L. and Melchiorri, C. (2008). *Trajectory planning for automatic machines and robots* (Berlin Heidelberg: Springer)

Ryan, R. M., Connell, J. P., and Plant, R. W. (1990). Emotions in nondirected text learning. *Learning and Individual Differences* 2, 1–17. doi:10.1016/1041-6080(90)90014-8
